# A Practical Treatise on the Diseases of Children

**Published:** 1853-12

**Authors:** 


*A Practical Treatise on the Diseases of Children.* By D. Francis Condie, M. D., Secretary of the College of Physicians, etc., etc. Philadelphia: Blanchard & Lea.

This is the fourth edition of this deservedly popular treatise. During the interval since the last edition, it has been subjected to a thorough revision by the author; and all new observations in the pathology, and therapeutics of children, have been included in the present volume.

Among the merits of this work we consider the first chapter "on the hygienic management of children," as pre-eminent. When, some years ago, we perused it in an earlier edition, we were struck with the common sense and good judgment which had dictated it. The directions commence with the management of the new-born infant, and give full details of the washing and dress, the air, temperature, food, and sleep, most conducive to the well-being of the new arrival. We are not aware of any other source where the young practitioner will find this subject so fully and sensibly treated.

Dr. Condie's volume has grown in size somewhat since the date of our first acquaintance with it. This growth it owes, not so much to changes, as additions. In the development of pathology the details are full and intelligible, though we think that they lack that exactness and conciseness, which mark the work of West on Diseases of Children. The fault of Dr. West's treatise is (to our sense) the heroic nature of the treatment recommended. From this fault (if such it be) Dr. Condie is not entirely free. We notice occurring at very short intervals, and in some very trivial affections, a certain prescription of calomel, ipecac, and hyosciamus, which seems to be a favorite of the author. Blood-letting also figures largely in the programme of remedial measures, and we were somewhat startled to find it so freely recommended in certain zymotic diseases, as scarlatina, and rubeola.

But this is a matter in which Dr. Condie does not stand alone. Our best standard work on Practice, (that by Watson, edited by Condie,) is liable to the same objection, and we have already intimated that Dr. West is even more open to this accusation. Yet we do not know of any safer or more complete work than this of Dr. Condie. Physicians do not rely upon books for their ideas of treatment. Blood-letting, which is so largely recommended by authors on diseases of children, is in fact ignored in practice. The phlebotomy of an infant is practically as rare an operation as that for tying the carotid; and while this holds true, we can see but little harm in the instruction of authors to the contrary. The free use of antimony and mercurials is also contra-indicated by the delicate organization of children, and their extreme susceptibility to impressions on the primæ viæ.

It is a hard matter for an author to break out of this time-honored routine of heroic remedies; and we are not surprised to find that so able a writer even as Dr. Condie, is not free from this influence. As we said before, we do not know of a better book on Diseases of Children, and to a large part of its recommendations we yield an unhesitating concurrence.

For sale by DERBY, ORTON & MULLIGAN. S. B. H.

